# Molecular Genetics Enhances Plant Breeding

**DOI:** 10.3390/ijms24129977

**Published:** 2023-06-09

**Authors:** Andrés J. Cortés, Hai Du

**Affiliations:** 1 Corporación Colombiana de Investigación Agropecuaria AGROSAVIA, C.I. La Selva, Km 7 vía Rionegro—Las Palmas, Rionegro 054048, Colombia; 2 College of Agronomy and Biotechnology, Chongqing Engineering Research Center for Rapeseed, Southwest University, Chongqing 400000, China; 3 Integrative Science Center of Germplasm Creation in Western China (Chongqing) Science City and Southwest University, of Agronomy and Biotechnology, Southwest University, Chongqing 400000, China; 4 Engineering Research Center, South Upland Agriculture, Ministry of Education, Chongqing 400000, China

Human-driven plant selection, a practice as ancient as agriculture itself, has laid the foundations of plant breeding and contemporary farming [1]. The principles of classical breeding still comprise the nucleus of modern crop science and agricultural production [2]. Recent unthinkable methodological achievements in molecular screening, genome sequencing, ‘omic’ technologies, trans-genesis, and computational power have advanced disciplinary boundaries in order to revolutionize the field of varietal development and its broader connections with related disciplines. These advances have opened up new trans-disciplinary arenas in which classical plant improvement intersects with genomics, molecular biology, biotechnology, and bioinformatics [3]. Yet, the factual potential of these interplays is often disregarded by prevailing atomized views and skepticism, both of which penalize the dawn of emerging properties and their ultimate deployment into framers’ fields [4]. Therefore, this second Special Issue of IJMS on “Molecular Genetics and Plant Breeding” exemplifies through 31 inter-disciplinary works (https://www.mdpi.com/journal/ijms/special_issues/gene_plant_breading_2nd, accessed on 6 June 2023) the ways in which disruptive molecular research opens up innovative pathways for the exploration, transformation, and utilization of crop biodiversity in order to improve essential breeding targets and replenish the genetic potential of the cultivated genepools in order to achieve greater yield and sustainability [5].

## 1. Marker-Guided Pre-Breeding

Natural genetic variation continues to constitute a major source of crop innovation, and its exploration remains a key milestone in molecular pre-breeding. This Special Issue provides some insights in this regard by presenting a series of molecular characterizations of diverse germplasm (Table 1). For instance, López-Hernández and Cortés [6] assessed the scale and determinants of somaclonal coding diversity in mint (*Mentha* spp.), introduced to the northern Andes, using RNA-seq on 29 clonally propagated accessions. The authors found a single genetic cluster for *M.* × *piperita*, and three clusters for *M. spicata*, suggesting two independent introductions of the latter.

Meanwhile, marker-assisted pre-breeding is underdoing a transformation from the method of classically screening crop genepools to the assessment of their associated antagonistic biotic agents. For example, Choi et al. [7] elucidated the molecular phylogenetic origin of seven strains of soybean mosaic virus (SMV) that had been sampled from 150 *Glycine max* L. accessions. The use of RNA-seq and 143 SMV available genomes enabled concluding that recombination and plant hosts drive the genetic diversity of SMV.

**Table 1:** Compilation of 31 studies as part of this second Special Issue of IJMS on “Molecular Genetics and Plant Breeding”. Four of the contributions were modern literature reviews (first rows).

Species	Goal	Sampling	Genotyping	Key Finding	Reference
Reviews					
Cruciferous crops and *Plasmodiophora brassicae*	^†^ Review clubroot control methods and breeding for resistant cultivars	Cruciferous crops	Resistance loci and R genes	Resistance loci offer feasible strategies for resistance breeding	Zhang et al. [8]
Various	^†^ Review functionality of *MYB* genes in plant roots	Plant roots	*MYB* genes	*MYB* gene functionality spans responses from biotic to abiotic stresses	Chen et al. [9]
Various	^†^ Review applications of miR393 for plant development and stresses	Various	miR393 targeting TIR1 and AFB auxin receptors	miR393 assists plant responses to biotic and abiotic stresses	Jiang et al. [10]
Various	^†^ Review the applications of AI in crop breeding	Various	AI and “omics” tools	Integration of AI into “omics” tools needed for crop-improvement	Khan et al. [11]
**Germplasm molecular characterization**					
Mint (*Mentha* spp.)	º Assess somaclonal coding diversity in mint at the northern Andes	A total of 29 clonally propagated mints from the northern Andes	RNA-seq	One and three clusters found for *M. × piperita* and *M. spicata*	López-Hernández and Cortés [6]
Soybean mosaic virus (SMV)	º Elucidate the molecular phylogenetic origin of SMV strains	A total of 7 SMV from 150 different soybean germplasm	RNA-seq and 143 SMV available genomes	Recombination and plant hosts drive the genetic diversity of SMV	Choi et al. [7]
Viromes of pepper *Capsicum annuum*	º Examine the viromes of 15 pepper cultivars through RNA-seq	A total of 15 pepper (*Capsicum annuum* L.) cultivars	RNA-seq of pepper viromes	First viromes in 15 major pepper cultivars through RNA-seq	Jo et al. [12]
**Genetic mapping**					
Rice (*Oryza sativa*)	^‡^ Infer the genomic bases (QTLs) of germination under cold conditions	One hundred and twenty lines of the CNDH population	778 SSR markers	Four QTLs and 41 genes were recovered, and 25 were qRT-PCR tested	Kim et al. [13]
Sesame (*Sesamum indicum*)	^‡^ Disclose the genomic basis (GWAS) for lignan lignin biosynthesis	410 accessions	WGR: 5.38 M SNPs and 1.16 M InDels	*SiNST1* is a target gene for the molecular breeding of lignans content	Dossou et al. [14]
Soybean (*Glycine max*)	^‡^ Reveal the genomic architecture (QTLs) for drought tolerance	A total of 160 RILs drought-tolerant ‘Jindou21’ × control ‘Zhongdou33’	WGR: 923,420 SNPs	Five QTLs may be useful for molecular marker-assisted selection	Ouyang et al. [15]
Soybean (*Glycine max*)	^‡^ Validate existing QTLs for MAS of pod-shattering tolerance	A total of 2 RIL families (154 and 153) + 102 varieties and elite lines	QTLs identified via WGR and 3 KASP markers	Recovered accuracy: 90.9% for RILs and 100% for varieties and lines	Seo et al. [16]
Wheat (*Triticum aestivum*)	^‡^ Reveal genomic basis (GWAS) for six traits in eight environments	In total, 509 European varieties (277) and breeding lines from the STH (232)	Total 13,499 DArTseq-derived SNP markers	A GWAS for heterosis revealed 1261 markers with significant effects	Mokrzycka et al. [17]
Wheat (*Triticum aestivum*)	^‡^ Reveal the genomic architecture (QTLs) of spike layer uniformity	A total of 300 RILs	Wheat 55 K SNP array: 53,063 SNPs and one KASP-marker	*QSlu.sicau-2B-2* is a target for MAS of spike-layer uniformity	Zhou et al. [18]
**Gene functional validation with expression analysis, RNA-seq, and/or other “omic” techniques**					
*Arabidopsis thaliana*	^+^ Validate the overexpression effects of *PIF4* for HSR	Columbia-0 (Col) ecotype	RNA-seq, qRT-PCR, and ChIP-qPCR	Overexpression of *PIF4* boosts basal thermotolerance	Yang et al. [19]
*Arabidopsis thaliana*	^+^ Characterize functionally *AtEAU1*-*AtEAU2* via RT-PCR	Columbia-0 (Col) ecotype	Two ABA-responsive EAR-motif-containing genes	*AtEAU1* and *AtEAU2* are novel repressors of ABA responses	Zhang et al. [20]
Cabbage (*Brassica oleracea*)	^+^ Explore the molecular mechanisms that enable ME via HS	Accession ‘01-88’	DNA methylation and miRNA	DNA methylation and miRNA interference regulate HS-induced ME	Kong et al. [21]
Chinese cabbage (*Brassica rapa*)	^+^ Map and validate *BrGOLDEN*, a dominant gene for carotenoid content	A total of 151 tri-crossed hybrids between ‘1900264’ and ‘1900262’	Full-length *BrGOLDEN* sequence and qRT-PCR	*BrGOLDEN* gives insights into the regulation of carotenoid synthesis	Zhang et al. [22]
Corn (*Zea mays*) and rice (*Oryza sativa*)	^+^ Validate PHO-encoding genes phylogenetically derived	Mo17 inbred line of *Z. mays*	PHO1- and PHO2- encoding genes + qRT-PCR	ABA could up-regulate the expression of both PHO1 and PHO2	Yu et al. [23]
Rapeseed (*Brassica napus*)	^+^ Identify and qRT-PCR test nitrate transporter 2 (*NRT2*) genes	Cultivars ‘Zhongshuang11’ and ‘Darmor-bzh’	A total of 31 and 19 *NRT2* genes respectively tested in ‘Zhongshuang11’ and ‘Darmor-bzh’	Candidates provided for functional *BnaZSNRT2s* studies	Du et al. [24]
Rapeseed (*Brassica napus*)	^+^ Validate *ARI* gene family functionality for agronomic traits	Varietal ‘ZS 11’	qRT-PCR of 39 *ARI* genes	Eight *BnARI* genes identified as candidates for key traits	Wahid et al. [25]
Rapeseed (*Brassica napus*)	^+^ Explore the functionality of Trihelix (*TH*) genes across Brassica species	Six Brassica species from the Brassica database (BRAD)	A total of 455 Trihelix (*TH*) genes available sequences and qRT-PCR	*BnaTH* genes are involved in response to drought, cold, and heat	Zhang et al. [26]
*Ribes nigrum*	^+^ Evaluate the molecular mechanisms related to BRV tolerance	Cultivar Aldoniai	RNA-seq	Novel transcripts for breeding BRV-tolerance are provided	Mažeikienė et al. [27]
Sacha Inchi (*Plukenetia volubilis*)	^+^ Recover transcriptomic and proteomic profiles of seeds at two stages	Three-year-old Peruvian trees introduced to South China	RNA-seq and iTRAQ	The study enables further research and utilization of RIPs	Liu et al. [28]
Sorghum (*Sorghum bicolor*)	^+^ Validate *DOF* genes functionality for starch biosynthesis	Cultivar ‘BTx623’	qRT-PCR of 30 *DOF* genes	*SbDOF21* acts as a key master regulator for starch biosynthesis	Xiao et al. [29]
Wheat (*Triticum aestivum*)	^+^ Characterize a new *Q* allele (*Q^c5^*) for compact spikes and good bread	Cultivar “Roblin”	*Q* gene and its new allele *Q^c5^*	*Q^c5^* boosts bread-making quality by repressing *SPR*	Guo et al. [30]
**Trans-genesis targeting inter-specific standing variation for validation purposes**					
Pitaya (*Hylocereus monacanthus*)	^§^ Map and qRT-PCR validate NAC candidate genes	Cultivar ‘Hongguan No. 1’	A total of 64 NAC TFs (from *HuNAC1* to *HuNAC64* genes)	*HuNAC20* and *25* from Pitaya confer cold tolerance in *Arabidopsis*	Hu et al. [31]
Woody resurrection plant (*Myrothamnus flabellifolia*)	^§^ Overexpressed *MfWRKY40* in *Arabidopsis* for abiotic stress roles	*M. flabellifolia* and *A. thaliana* Columbia-0 (Col) ecotype	WRKY TFs (i.e., early dehydration-induced gene *MfWRKY40*)	*MfWRKY40* confers tolerance to drought and salinity in an *Arabidopsis*	Huang et al. [32]
**Trans-genesis targeting intra-specific standing variation**					
Rapeseed (*Brassica napus*)	^λ^ Assess homozygous transcript overexpression lines for *γ-TMT*	Cultivar ‘Zhongshuang11’	Gene γ-Tocopherol methyltransferase (*γ-TMT*)	Feasible to genetic engineer *γ-TMT* for salt tolerance breeding	Guo et al. [33]
Soybean (*Glycine max*)	^λ^ Silence *GmBIR1* by BPMV-VIGS and explore the phenotypes	Cultivar ‘Williams 82’	*BAK1*-interacting receptor-like kinase (*GmBIR1*)	*GmBIR1* is a negative regulator of immunity in soybean	Liu et al. [34]
**Mutagenesis targeting intra-specific de novo mutations**					
Wheat (*Triticum aestivum*)	^Ψ^ Characterize mutant *Q* alleles for grain yield and grain protein	Wheat cultivar ‘Shumai482’ and its *S-Cp*1-1 mutant	Two new *Q* alleles (*Q^s1^* and *Q^c1^-N8*) obtained via mutagenesis	New *Q* alleles offer novel germplasm relevant to wheat breeding	Chen et al. [35]
**Introgression breeding targeting inter-specific standing variation**					
Rice (*Oryza sativa*)	^θ^ Breed for heat tolerance in rice via introgressed sorghum ancestry	Rice restorer line ‘R21’ and recipient restorer line Jin ‘Hui 1’	WGR	The sorghum introgression in line ‘R21’ confers mobile heat tolerance	Zhang et al. [36]

Table is arranged top-down by research goals, species, and citations. Studies are clustered by goals as follows: ^†^ for reviews; º for germplasm molecular characterization; ^‡^ for genetic mapping; ^+^ for gene functional validation with expression analysis, RNA-seq and/or other omic techniques; ^§^ for trans-genesis targeting inter-specific standing variation for validation purposes; ^λ^ for trans-genesis targeting intra-specific standing variation; ^Ψ^ for mutagenesis targeting intra-specific de novo mutations; and ^θ^ for introgression breeding targeting inter-specific standing variation. Abbreviations are as follows. ABA: abscisic acid; AFB: auxin signaling F-box; ARI: Ariadne proteins of ring-between-ring (RBR) finger protein subfamilies; BPMV-VIGS: bean-pod-mottle-virus-induced gene silencing; BRV: mite-transmitted blackcurrant reversion virus; CNDH: Cheongcheong Nagdong double haploid; DOF: C2-C2 zinc finger domain, EAR: ethylene-responsive element-binding-factor-associated amphiphilic repression; AI: artificial intelligence; GWAS: genome-wide association study; HS: heat shock; HSR: heat stress response; KASP: Kompetitive allele-specific PCR; MAS: marker-assisted selection; ME: microspore embryogenesis; PIF4: phytochrome interacting factor 4; PHO: starch phosphorylase; QTL: quantitative trait loci; RILs: recombinant inbreed lines; RIP: ribosome-inactivating protein; SNP: single-nucleotide polymorphism; SPR: storage protein repressor; SSR: simple sequence repeat; STH: Plant Breeding Strzelce, TIR1: transport inhibitor response1; TF: transcription factors; WGR: whole-genome re-sequencing.

Similarly, Jo et al. [12] systematically examined the viromes of 15 pepper (*Capsicum annuum* L.) cultivars through RNA-seq, enabling a high-throughput identification of the principal viromes present in commercially important pepper genotypes. As a promising perspective, these initial characterizations of natural variation may unleash a further potential when coupled with explicit estimates of their genomic bases, i.e., the genetic determinants of trait variation.

As such, genetic mapping arises as a key step in the pre-breeding pipeline that enables the genetic architecture of key traits to be disclosed, and ultimately provides candidate markers and genes for further marker-guided applications, such as parental screening, marker-assisted selection [37], and gene editing [38]. In this regard, and as part of this Special Issue, Kim et al. [13] genotyped 120 double haploid rice lines with 778 SSRs in order to reconstruct the genetic basis of germination under cold conditions. The authors managed to retrieve 4 QTLs and 41 genes, 25 of which were validated via qRT-PCR. Similarly, Dossou et al. [14] characterized 410 sesame (*S. indicum*) accessions using WGR, and performed GWAS for lignan–lignin biosynthesis. The team found that *SiNST1* is a major target gene for the molecular breeding of lignan content. In soybean, Ouyang et al. [15] and Seo et al. [16] performed WGR of RIL families with ca. 155 genotypes each. The teams, respectively, identified five QTLs for drought tolerance, and validated accuracies above 90% for existing QTLs for pod-shattering tolerance. Finally, Mokrzycka et al. [17] and Zhou et al. [18], respectively, genotyped 509 wheat accessions and 300 RILs with 13,499 DArT-SNPs and a 55 K SNP array. The authors discovered 1261 candidate markers for six agronomical traits, and the *QSlu.sicau-2B-2* MAS target for spike layer uniformity. A common denominator across all genetic mapping exercises is the need for a further validation of the associated markers [39] before any downstream plant improvement application. After all, they are prone to displaying inflated rates of false positives due to multiple comparison testing, population stratification, and intrinsic redundancy from linkage disequilibrium (LD) [40].

Marker validation often requires that a battery of downstream analyses be performed. The first step required is narrow mapping of the causal variants using target genotyping across extended panels. A second, more experimental approach involves examining the expression profiles of flanking candidate genes in terms of congruency and stability. Several teams pursued this goal as part of the current Special Issue. For example, Yang et al. [19] and Zhang et al. [20] used the Columbia-0 (Col) *A. thaliana* ecotype to validate the functional roles of *PIF4* and *AtEAU* (*1* and *2*) genes, respectively, through transcriptomic tools as boosters and repressors of basal thermotolerance and ABA response. Meanwhile, Kong et al. [21] and Zhang et al. [22], respectively, implemented epigenomics and qRT-PCR in cabbage (*B. oleracea*) and Chinese cabbage (*B. rapa*) to study the DNA methylation footprint during microspore embryogenesis induced by heat shock, and the functional role of *BrGOLDEN* in carotenoid biosynthesis. Interestingly, rapeseed (*B. napus*) was a widely considered study system by Du et al. [24], Wahid et al. [25], and Zhang et al. [26], who used qRT-PCR to corroborate the functionality of *BnaZSNRT2s*, *BnARI,* and *BnaTH* genes for nitrogen uptake, agronomical trait regulation, and abiotic stress tolerance, respectively. Other studies focused on key crops for food security. For example, while also considering the ABA pathway, Yu et al. [23] used qRT-PCR to demonstrate the up-regulation of PHO-encoding genes by ABA in maize and rice. On the other hand, Xiao et al. [29] validated *SbDOF21* as a key regulator for starch biosynthesis in sorghum, and Guo et al. [30] characterized a new *SPR*-repressor *Q* allele (*Q^c5^*) for compact spikes and bread quality in wheat. The other studies which stand out are those by Mažeikienė et al. [27] and Liu et al. [28], who brought RNA-seq technology to the new promissory crops *Ribes nigrum* and Sacha Inchi (*Plukenetia volubilis*). Each team was able to provide transcriptomic resources to breed for tolerance to the BRV virus [27] and better comprehend the expression profiles of seeds at two different developmental stages [28].

Finally, the works by Hu et al. [31] and Huang et al. [32] utilized trans-genesis to move beyond the species boundary and validate in an *Arabidopsis* background candidate genes for cold (*HuNAC20* and *25*) and osmatic (*MfWRKY40*) stress tolerance, respectively, from two other exotic crops, namely, the cactus-fruit Pitaya (*Hylocereus monacanthus*) [31] and the woody resurrection plant (*Myrothamnus flabellifolia*) [32]. Despite these advances in germplasm characterization, genetic mapping, and functional validation, moving forward, elite cultivars are beginning to benefit from embracing genomic-enabled breeding.

## 2. Molecularly Enabled Breeding

Mobilizing germplasm’s allelic novelty, leveraging marker genetic mapping, and functionally corroborating candidate genes to customize crops requires the modernization of classical plant breeding with phenomic and genomic tools [2]. Perhaps one of the promptest strategies for bridging long breeding cycles is trans-genesis. When it targets standing variation, segregating within the same species genepool, it simply provides a shortcut to the time-consuming alternative of recurrent backcrossing [37]. However, it also allows customizability for de novo variation. This Special Issue compiles an exquisite repertoire in this regard. For instance, Guo et al. [33] illustrated, once again in rapeseed, that it is feasible to genetically engineer *BnaC02.TMT.a*, a *γ-TMT* paralogue, for salt tolerance breeding via the use of homozygous transgenic lines. Similarly, Liu et al. [34] silenced *GmBIR1* with BPMV-VIGS to demonstrate that it is a negative regulator of immunity in soybean. Finally, Chen et al. [35] went one step further and explored mutagenesis as a reliable source of de novo allelic variation. The authors characterized mutant *Q* alleles (*Q^s1^* and *Q^c1^-N8*) for grain yield and grain protein in the wheat cultivar ‘Shumai482’ and its *S-Cp*1-1 mutant [35], complementing the results achieved by Guo et al. [30] in terms of compact spikes and bread quality. In spite of these exciting results achieved for the development of trans-genesis, classical intercrossing has not lost validity as a breeding strategy, but rather it has been permeable to the latest genomic advances, such as marker-assisted backcrossing (MABC) [37], introgression breeding, and genomic-assisted recurrent selection [41].

Inter-specific introgression breeding, in particular, aims to break species boundaries to pyramid exotic variation from one species to elite commercial varieties from the other species [37]. However, inter-specific crossing conveys two main challenges, which are species incompatibility and polygenic trait variation. Therefore, coupling hybrid breeding with bridge genotypes and guiding molecular markers unleashes novel opportunities to improve the chances of success, pace, and precision of the target introgression. Such utility has been reinforced in rice by Zhang et al. [36], who bred for heat tolerance by introgressed sorghum ancestry. The authors utilized the rice restorer line ‘R21’ and the recipient restorer line Jin ‘Hui 1’, and were able to demonstrate with WGR that the sorghum introgression in line ‘R21’ confers mobile heat tolerance. Gene editing may speed up rice breeding for abiotic stresses [42], too. Bean breeders are also seeing a similar development by GBS-genotyping 87 advanced lines with inter-specific ancestries between common bean (*P. vulgaris*) and tepary bean (*P. acutifolius*) for heat and drought tolerance across four environments in coastal northern South America [43]. The authors found 47 associated loci and 90 flanking candidate genes for molecular-guided downstream selection. Meanwhile, the team also detected suitable allelic variation within the candidate genes of tepary bean (*P. acutifolius*) that transcends the adaptive genepool of common bean (*P. vulgaris*) [44]. These studies demonstrate that the integration of genomic- and gene-based strategies can leverage inter-specific adaptive variation via bridge genotypes in order to deliver candidate introgressed lines for heat tolerance [43]. In certain cases, grafting could also provide a fast track to harness such species diversity [45]. These success stories that intermingle modern genotyping technology with classical intercrossing exemplify, against any intellectual skepticism, the factual applicability of the molecular breeding paradigm. Still, there remains room for further developments.

## 3. Perspectives

As molecular and phenomic data continue to pile up, modern analytical techniques must be embraced. Predictive breeding, reached throughout genomic-enabled selection [41] and machine learning [11] at the interface of the plant breeding triangle [46], confers a primary way forward capable of bringing together molecular biology and plant genetic paradigms [47]. In this regard, Khan et al. [11] reviewed applications of crop breeding in this Special Issue, calling for a better integration of AI with “omics” tools. Still, effective crop mobilization networks and open access data must be assured in order to build sufficiently reliable training datasets without sampling bias or over-fitting prediction [48].

Meanwhile, biotic and abiotic stresses are becoming more common, jeopardizing global food production. In this sense, another review within this collection by Zhang et al. [8] was more concrete in envisioning gene targets for biotic pressures, specifically that clubroot control methods in cruciferous crops could be harnessed with allelic variation at the R genes, which are in turn susceptible to be gene-edited, eventually. Similarly, the reviews by Chen et al. [9] and Jiang et al. [10] prospected gene-enabled abiotic stress responses by looking at the *MYB* genes in plant roots and the miR393 during plant development, respectively.

The method of screening the genetic determinants of the abiotic susceptibility, and its potential gene editing, should be supplemented by explicit in situ eco-physiological indices targeting specific stresses, performing potential ecological niche modeling (ENM) under present and future scenarios as part of climate vulnerability assessments, undertaking gap analyses of the available variation for pre-breeding, exploring genomic selection signatures of historic adaptation, and unpicking the genome–environment associations (GEA) of current niche preferences [3]. After all, tackling the climate crisis and agrobiodiversity loss in the process of addressing food security [5] demands that we harness trans-disciplinary sustainable enterprises that promote integrative agendas among the otherwise disentangled fields of in situ and ex situ conversation, physiology, ecology, molecular genetics, plant breeding [2], conservation, seed delivery [4], food policy [49], and marketing.

## Data Availability

For original datasets, please refer to the published articles [6,7,8,9,10,11,12,13,14,15,16,17,18,19,20,21,22,23,24,25,26,27,28,29,30,31,32,33,34,35,36] within the second Special Issue of IJMS on “Molecular Genetics and Plant Breeding 2.0” (https://www.mdpi.com/journal/ijms/special_issues/gene_plant_breading_2nd, as accessed on 6 June 2023).
